# Methane hydrate emergence from Lake Baikal: direct observations, modelling, and hydrate footprints in seasonal ice cover

**DOI:** 10.1038/s41598-019-55758-8

**Published:** 2019-12-18

**Authors:** N. G. Granin, I. A. Aslamov, V. V. Kozlov, M. M. Makarov, G. Kirillin, D. F. McGinnis, K. M. Kucher, V. V. Blinov, V. G. Ivanov, I. B. Mizandrontsev, A. A. Zhdanov, A. S. Anikin, M. N. Granin, R. Yu. Gnatovsky

**Affiliations:** 1 Limnological Institute, Siberian Branch of Russian Academy of Science, (LIN SB RAS), Irkutsk, 664033 Russia; 2 Matrosov Institute for System Dynamics and Control Theory of Siberian Branch of Russian Academy of Sciences (ISDCT SB RAS), Irkutsk, 664033 Russia; 3 Department of Ecohydrology, Leibniz-Institute of Freshwater Ecology and Fisheries (IGB), Berlin, 12587 Germany; 4 Aquatic Physics Group, Department F.-A. Forel for Environmental and Aquatic Sciences (DEFSE), Faculty of Science, University of Geneva, Geneva, 1211 Switzerland

**Keywords:** Limnology, Limnology

## Abstract

This paper provides a novel report of methane hydrates rising from bottom sediments to the surface of Lake Baikal, validated by photo and video records. The ascent of hydrates in the water column was confirmed by hydroacoustic data showing rising objects with velocities significantly exceeding the typical speeds (18–25 cm s^−1^) of gas bubbles. Mathematical modelling along with velocity and depth estimates of the presumed methane hydrates coincided with values observed from echograms. Modelling results also showed that a methane hydrate fragment with initial radius of 2.5 cm or greater could reach the surface of Lake Baikal given summer water column temperature conditions. Results further show that while methane bubbles released from the deep sedimentary reservoir would dissolve in the Lake Baikal water column, transport in hydrate form is not only viable but may represent a previously overlooked source of surface methane with subsequent emissions to the atmosphere. Methane hydrates captured within the ice cover may also cause the formation of unique ice structures and morphologies observed around Lake Baikal. Sampling of these ice structures detected methane content that exceeded concentrations measured in surrounding ice and from the atmosphere demonstrating a link with the methane transport processes described here.

## Introduction

Methane production in inland freshwater bodies is an intensive area of ongoing research due to the greenhouse potential of methane^1^. Inland freshwater bodies make significant contributions to the atmospheric methane budget^2^ (6–16% of total natural methane emissions). Because lakes are major contributors to the freshwater carbon cycle, their methane production and release dynamics require more precise understanding and quantification^3^. A common assumption regarding methane in lakes is that it is produced primarily by anoxic sediments and/or in the deep-water column^4,5^. Some of this methane can reach the lake surface due to convection during lake turnover, but water column oxidation significantly reduces the dissolved methane concentration such that only a small fraction reaches the atmosphere^6,7^. Alternatively, methane bubbles originating from the sediment may ascend to reach the atmosphere, but this transport mechanism depends on the initial size of the bubble and its release depth^8^. Several recent studies have also suggested that methane production and transport in lakes can be highly variable and may include significant methane production within the oxygen-rich upper water column^5,9^. These results demonstrate that methane release from well-oxygenated lakes may not always be negligible^10^.

As the world’s deepest lake, Lake Baikal is the only lake known to host solid phase methane (methane hydrates). Formation of methane hydrates is facilitated by high pressure and low temperatures in abyssal areas^4,11^. The possible presence of methane hydrates in bottom sediments of Lake Baikal was initially postulated from results collected by a joint expedition of the Limnological Institute and VNIIGAZ (Moscow) in 1978^12^. Seismic profiles^13^ further indicated the presence of methane hydrates but unambiguous evidence was not obtained until drilling in deep water areas of Lake Baikal in 1996^11^. In recent decades, several expeditions have studied methane hydrates in near-bottom sediments^14,15^ as well as gas seeps in both shallow and deeper areas of the lake^16–18^.

Hydrates embedded within lake bottom sediments can be mobilized by water level variations, earthquakes, or other disturbances in water column pressure^19–22^. Recently, unique ring structures observed within Lake Baikal ice have been interpreted as resulting from methane hydrate release^23^. Earthquakes have been shown to influence the formation and decomposition of methane hydrates in near-bottom sediments of Lake Baikal^24,25^. The question of whether sediment-formed methane hydrates can reach the lake surface once they break away however remains unresolved. Brewer *et al*.^26^ performed experiments on the controlled transit of methane hydrates through the marine water column (Pacific Ocean) using a remotely operated underwater vehicle (ROV). This research showed that hydrate fragments of 8–10 cm diameter rising from 800 m depth can reach the surface and deliver methane to the atmosphere. Zhang and Xu’ll^27^ performed numerical modeling of a methane-seawater system along a T-P-depth profile similar to that of Blake Ridge (east of the U.S. South Carolina coast). These researchers found that only methane hydrate fragments larger than 9 cm in radius could rise through a 530 m water column and reach the ocean surface. Methane hydrates ascending to the surface of Lake Baikal have not been previously reported or systematically studied.

**Figure 1:**
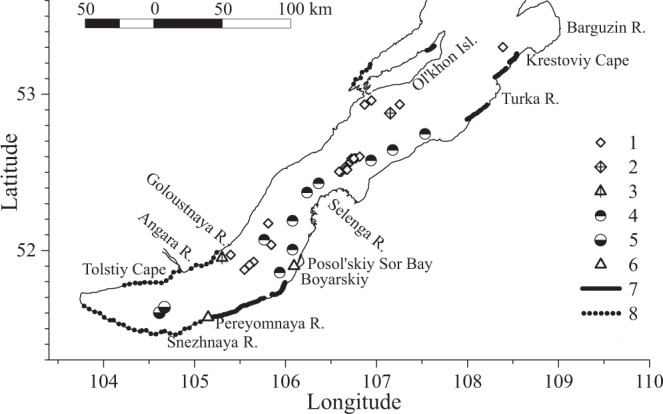
Map of the central and southern Lake Baikal basin including (1) methane hydrates detected in the surface layer of bottom sediments, (2) “St.Petersburg” mud volcano, (3) “Stupa” gas seep, (4) kolobovnik reported by Granin and Granina^30^, (5) kolobovnik investigated in 2014, (6) sopki investigated in 2017 and 2018, (7) shore areas with sopki in 2018, (8) shore areas without sopki in 2018.

Lake Baikal is covered by ice from January until May. If methane hydrates reach the lake surface, their traces can appear in the form of altered surface ice structure and/or increased methane concentrations in ice. Two distinctive forms of surface ice found around Lake Baikal may record the ascent of methane hydrates and their interaction with the lake’s ice cover. One form of ice is referred to as”kolobovnik”, a local name for ice containing large amounts of lumpy, granular ice with a rough surface with ice-balls from a few centimeters to 0.2–0.5 m in diameter, frozen in the ice cover. Sediment particles may occur within the ice^28,29^. Historically, kolobovnik has been observed in different pelagic areas of the lake (Fig. 1, black/white circles) where it has been interpreted as forming in association with methane hydrates^30^. In 2002, kolobovnik was discovered in the center of south Lake Baikal. Analysis of biogenic-terrigenous sedimentary particles within the ice (the concentration of which was less than 0.8 g L^−1^) suggested that they were derived from the littoral zone of the lake, but unfortunately methane analyses were not conducted^31^.

Another ice form potentially associated with methane hydrates is locally referred to as “sopki’. Sopki appears more frequently on the east side of the lake but sometimes occurs along the northwest shore^32^. Tsurikov described sopki as cone-shaped ice hillocks surrounding a hollow area or an opening that faces toward the lake. The structures are described as resembling a tent in shape^32^. Sopki may form as isolated units, in groups, or in series (a “mountain range”) with the largest features reaching heights of up to 6 m. Obvious holes present in sopki differentiate them from typical coastal ice mounds formed by water waves and splashing. However, sopki and kolobovnik morphologies and formational mechanisms have not been systematically described in the scientific literature.

This study utilized visual observations and acoustic echosounding surveys to document the ascent of methane hydrates through the Lake Baikal water column. To estimate the minimum size of hydrate capable of reaching the lake surface, we developed a numerical model and analyzed theoretical methane hydrate transformation during ascent from bottom sediments to the lake surface. We also report novel data demonstrating increased methane concentrations in kolobovnik and sopki ice structures. Data support the interpretation that these ice features could form from methane hydrates ascending to the lake surface. In contrast to bubbles or dissolved methane, methane hydrates are more stable in the water column to transport methane from Lake Baikal sediment to the atmosphere. The ascent of the methane hydrates through the water column thus represents a potentially important pathway linking methane produced in deep lacustrine sediments to the surface water methane^4,14^.

## Methods

Study site and field methods. Lake Baikal (N53°13′00″ E107°45′00″) is the deepest (max. depth 1642 m) and most voluminous (23.6 × 10^3^ km^3^) freshwater lake in the world. Located near the center of the Siberian high-pressure system, the lake is subject to strong cooling in winter, which results in a 3–5 month ice-covered period^33^. Lake Baikal is the only freshwater lake where methane hydrates are present in deep-bottom sediments^11^ including subsurface areas^4^ (beneath the sediment – water interface).

An acoustic survey was performed in the winter of 2013 with a Furuno FCV-1100 echosounder equipped with two channels (28 and 200 kHz) scanning a 24° and 8° field of view, respectively at up to 3 kW power. The pulse duration was 3 ms (which corresponds to a 4.2 m distance assuming 1425 m s^−1^ as the speed of sound in water). The pulse repetition frequency was 0.2 Hz. A hole was excavated in the ice to position the transducers above an area of gas seeps previously detected at the lake bottom. The transducers were mounted on a specially designed rig that ensured their axis remained vertical. A CTD (Conductivity-Temperature-Depth) probe SBE-25 (SeaBird USA) was used to performed temperature and conductivity profiles for later processing of acoustic sounding data and use in modelling (temperature accuracy: ±0.002 °C; conductivity accuracy: ±0.01 µs cm^−1^ or ±0.02 mg kg^−1^ of the sum of ions). An Infinity EM 2D electromagnetic current meter (JFE Advantech Co., Ltd.) was used to measure the current velocity profile (velocity range ±5 m s^−1^, resolution 0.02 cm s^−1^, accuracy ±1 cm s^−1^). Current speed measurements were averaged for 2 minutes at each depth as determined by rope markers.

Methane concentrations in the ice cover were measured from ice cores retrieved using saws. Each ice core was cut into 5–10 cm sections from top to bottom. Sections were weighed and then submerged in saturated brine to melt within a closed vessel. A hose with a clamp and syringe was attached to the vessel to collect the gas from air bubbles included in the ice. After ice melting, the volume, temperature and pressure of gas obtained from each section was determined and then the measured volume was recalculated to standard temperature and pressure conditions (20 °C and 101.325 kPa)^34^. The volume of the section was determined according to the observed change in volume of the brine solution. The collected gas was then injected into a gas chromatograph (ECHO-EW with Flame-ionization detector, Novosibirsk, Russia). Methane concentrations in the gas were determined as parts per million per mole with 5% precision. Identification of methane was performed by its retention time and quantified using a set of gas standards with a pre-defined methane content of 0, 3.94, 8.37 and 10000 ppm (Pure Gases LLC, Novosibirsk, Russia).

### Mathematical modeling

A numerical model was constructed to describe phase transitions, velocity of ascending methane hydrates, and kinetics of hydrate decomposition. The model assumes a single hydrate fragment rising in an unconfined volume of water at decreasing pressure *p* and increasing temperature *T*. Decomposition and dissociation on the solid surface results in formation of an intermediate layer (an “ice crust”; see Supplementary Fig. S1)^27,35^. This layer has a complex heterogeneous structure consisting of porous ice (snow) saturated with gas, fragments of decomposing hydrate crystals, and possible water^27,36–41^. The model treats the methane hydrate, ice (snow), and water as incompressible. A basic description of the modelling approach is given below while the online Supplementary methods section gives a more detailed description.

The model assumes an ascending hydrate fragment as a sphere of radius *a*_*h*_ encapsulated within an ice crust of thickness *δ*_*i*_ = *a*_*i*_ – *a*_*h*_, where *a*_*i*_ is the equivalent spherical radius of the outside border of the ice crust. The equation for determining the ascending speed *w*(*t*) of the spherical body is obtained from the balance of momentum for a solid body with variable mass:

1 $$({m}_{h}+{m}_{w})\frac{dw}{dt}={f}_{A}-{f}_{T}-{f}_{W}-{f}_{M}$$

where *m*_*h*_ is the mass of hydrate, *m*_*w*_ is the mass of encapsulating water, and *f*_*A*_, *f*_*T*_, *f*_*W*_, *f*_*M*_ are buoyancy, gravity, hydrodynamic resistance, and reactive force, respectively. The forces acting on the hydrate fragment (right side of Eq. 1) depend on the size and density of the ascending sphere. The latter are determined from equations of heat transfer across the ice crust assuming that temperatures at the water-crust and crust-hydrate boundaries are fixed at the melting point and the equilibrium temperature of the respective methane hydrate phase transformations. The model assumes a linear radial temperature profile within the ice crust. The resulting model thus becomes a system of ordinary differential equations describing the evolution of four variables: vertical position *z*(*t*), rising velocity *w*(*t*), and the inner and outer radii of the ice crust denoted as *a*_*h*_(*t*) and *a*_*i*_(*t*), respectively. The system can be solved numerically for a given set of initial conditions, namely temperatures and pressures measured from the environment. The model could also inversely estimate the initial radius of a hydrate and thermal properties of the ice crust using experimentally observed ascending speeds of hydrates as inputs.

## Results

### Ascending methane hydrates: direct observations and model estimates

**Figure 2:**
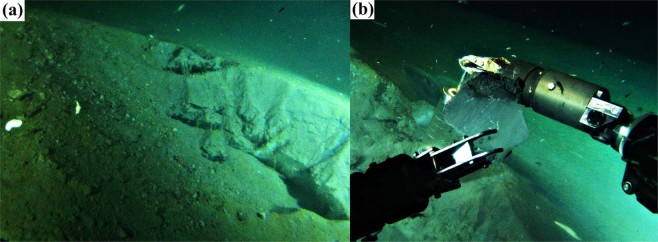
Photograph of exposed methane hydrates in lake sediment (**a**) and a methane hydrate fragment held by the manipulator arm. (**b**) The site shown is located the middle region of Lake Baikal, an area close to the St. Petersburg mud volcano at a depth of 1350 m.

**Figure 3:**
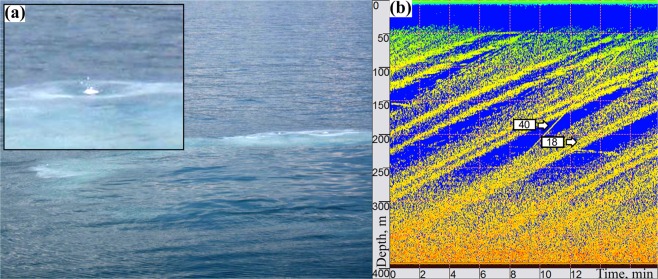
(**a**) Methane hydrates detected at the lake surface. Inlay: Close-up of exploding methane hydrate. (**b**) Echogram of gas bubbles rising with speeds of 18 cm s^−1^ and methane hydrate rising with speed of about 40 cm s^−1^.

To date, methane hydrates have been detected in the surface layer of bottom sediments at 21 stations (Fig. 1, diamonds) in the south and middle basins of Lake Baikal^14,15^. Some methane hydrate occurrences coincide with deep-water gas seeps (flares)^16,17^ but direct observations of methane hydrates are still rare. A 2009 expedition of the deep-water submersible MIR^17,41^ made a visual detection of methane hydrates at 1350 m water depth in sediments near a mud volcano referred to as St. Petersburg (Fig. 1, diamond with cross). We identified methane hydrates layers exposed within surface sediments (Fig. 2) and thus are potentially able to alight from the bottom due to mechanical disturbance. The methane hydrates appeared transparent with no significant inclusion of sediment. During 28 July 2013 sampling near a gas seep referred to as “Stupa” (depth approximately 500 m; see Fig. 1), we made a novel observation of methane hydrates emerging at the lake’s surface (Fig. 3). After retrieving a water sampling rosette, a few small (~10 cm) hydrate fragments ascended all the way to the surface of the lake where they subsequently exploded due to intense internal gas pressure.

**Figure 4:**
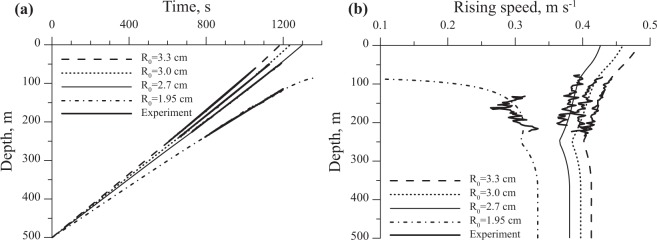
Estimated depths (**a**) and speeds (**b**) of ascending methane hydrates with different initial radius R_0_. Bold curves are the data obtained via hydroacoustic survey.

Acoustic surveys performed on 5 April 2013 from the ice surface above the Stupa gas seep allowed identification of ascending objects. A majority of these features were identified as gas bubbles ascending at speeds of about 18 cm s^−1^ (Fig. 3b). Surveys also detected four single sound-reflecting objects ascending at speeds of 38 to 43 cm s^−1^ over a three-hour period from 16:00 to 19:00 LT (see Supplementary Fig. S2, and Fig. 3b for an example). To determine whether these were fragments of methane hydrates, we inversely modeled an ascending hydrate (see Supplementary information online) using the trajectory and speed of the hydrate fragments obtained from echogram data. The CTD profiles provided water column physical property inputs (see Supplementary Fig. S1). For the measured temperature profile of the water column beneath the ice, the model estimated an upper stability limit for methane hydrates at equilibrium temperature *T* = 276.75 K and corresponding pressure *p = *3.72 × 10^6^ Pa or 372 dbar^17^. The water depth was 500 m. The model estimated the theoretical initial radius ${a}_{h}^{0}$ of the ascending hydrate fragment and effective thermal conductivity coefficient *k*_*ef*_ as: ${a}_{h}^{0}$ = 1.95, 2.7, 3.0, and 3.3 cm for the four particles detected and ${k}_{ef}=1.95$ (corresponding to an effective ice crust thermal conductivity ${\bar{\lambda }}_{ef}$ ranging from 0.2 to 0.6 W m^−1^ K^−1^). Estimated depths and velocities for ascending hydrate fragments of different radii agreed well with echogram data (Fig. 4).

The stability boundary for Structure I (KC-I) methane hydrate in freshwater containing only methane as the guest molecule occurs at depths of 360–380 m for a seasonal temperature range of 3.40 to 3.65 °C^16,37^. To determine the smallest possible initial radius ${a}_{h}^{0}$ necessary for hydrate fragments to ascend from this stability depth to the lake surface, we modeled particles of different initial radii ascending through different temperature profiles possible in the lake. Characteristic long-term average water temperature profiles ${T}_{l}={\phi }_{l}(z)$ for South Baikal occur in March (winter – presence of ice cover), August (summer – stratified water column with maximum temperatures at the surface), and November (isothermal conditions)^33^. Using these as inputs, the model estimated an initial radius ≥2.5 cm necessary for methane hydrates to ascend from the upper boundary of the hydrate stability zone to the surface of Lake Baikal.

### Hydrate footprints in the seasonal ice cover

#### Kolobovnik

**Figure 5:**
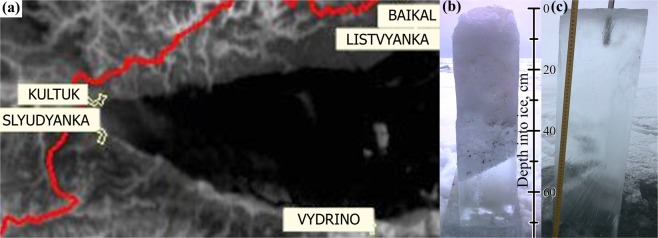
A satellite infrared image of Lake Baikal (southern basin) from the website sputnik.irk.ru on 30.01.2014 (**a**), photographs of ice core excavated from a kolobovnik ice field (**b**) and pure ice sampled from an area outside the kolobovnik ice field (**c**).

**Figure 6:**
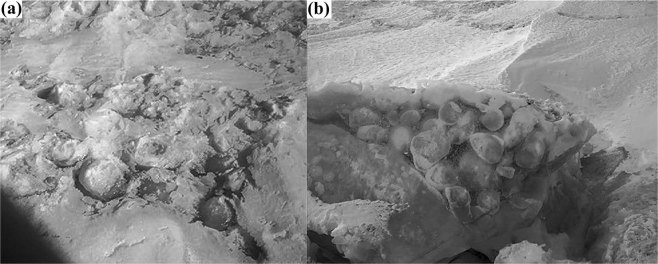
Undisturbed kolobovnik ice surface (**a**) and cross section of kolobovnik ice (**b**) from the south basin of Lake Baikal.

The porous and inhomogeneous structure of kolobovnik ice serves to diminish thermal conductivity relative to that of clear/normal ice. This effect reduces heat flow from water which makes it possible to differentiate the lower temperature of the kolobovnik ice in infrared satellite imagery. Infrared images of ice cover in 2014 show two fields of kolobovnik as discrete cold regions, one small and another larger region nearby (Fig. 1; Fig. 5a). Figure 6 shows undisturbed ice surfaces punctuated by kolobovnik structures and a cross section through kolobovnik ice. The spherical structures were relatively uniform in size and ranged from 10 to 20 cm in diameter.

**Figure 7:**
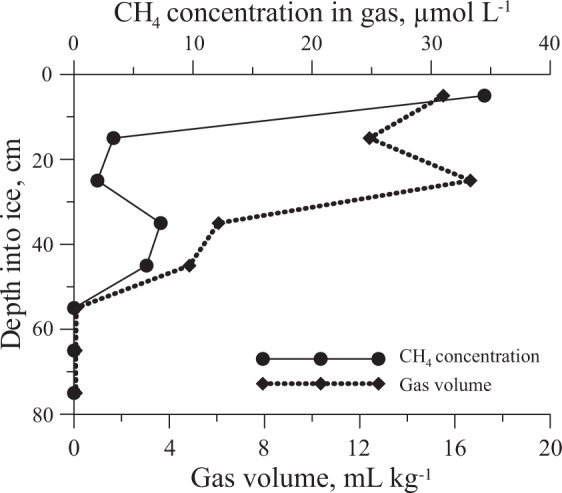
Methane concentrations from ice columns collected from kolobovnik ice (Fig. 5b) as a function of depth. Graph also shows gas volume (mL kg^−1^) within the ice column.

Ice cores were collected from the larger field of kolobovnik and surrounding areas (for background conditions) (Fig. 5b,c). Fig. 6b shows a clearly visible boundary between kolobovnik and clear background ice at about 50 cm depth. We analyzed gas content of the ice to test the hypothesis of a possible relationship between kolobovnik ice and emerging methane hydrates. Results showed that upper layers of ice were enriched in gases including methane. Gas volume decreased with depth from 15 to 0.2 mL kg^−1^ of ice (Fig. 7). Maximum methane concentrations of 35 μmol L^−1^ (780 ppm) occurred in the upper 10 cm of the ice. These values greatly exceeded atmospheric concentrations (about 85 nmol L^−1^ or 1.9 ppm). Gas concentrations then decreased to 2000 nmol L^−1^ (44 ppm) in the layer between 20–30 cm and increased to 8000 nmol L^−1^ in the layer between 30–40 cm. Below 55 cm, ice became clear and its gas volume did not exceed the 0.2 mL kg^−1^ detection limit of the analytical method used. Ice samples from areas surrounding the kolobovnik did not show any increased methane concentrations and were close to equilibrium with the atmosphere.

#### Sopki

**Figure 8:**
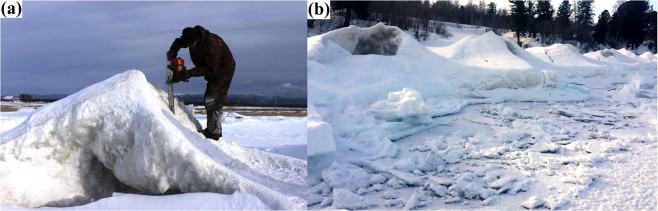
A photograph of a sopki structure on the western shore of Lake Baikal near Posol’sky Bay taken in March 2017 (**a**) and near the Pereyemnaya River in January 2018 (**b**).

**Figure 9:**
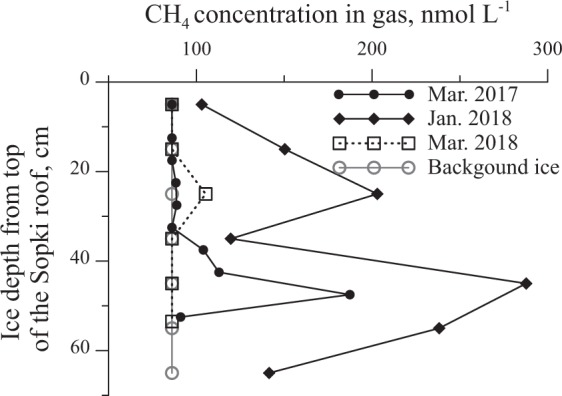
Methane concentrations measured from gas inclusions in the roof of a sopki structure sampled in 2017 and 2018. Data are also provided for clear background ice (not part of a sopki structure).

In 2017, sopki ice structures were observed on a strip of land separating the open water of Lake Baikal from Posol’skiy Sor Bay (Fig. 1, triangle; 8a). During the winter of 2018, we surveyed coastal areas of Lake Baikal and found sopki as indicated by bold lines in Fig. 1 (dotted lines indicate areas without sopki). In March 2017, an ice core was collected from the ice roof of one of the hills or tent structures. Analysis of gas extracted from the ice (Fig. 9) revealed a higher concentration of methane in the central area of the ice roof. Here, methane concentrations reached 200 nmol L^−1^ (4 ppm), a value which exceeds average values observed in the atmosphere by a factor of two. Sopki ice structures were also identified along the western shore of Lake Baikal, near Pereyomnaya River (51°34′51′′N 1105°16525′′E) on 11 January 2018. An ice core was collected from this site on 17 January (triangle in Fig. 1; Fig. 8b). Methane concentrations varied from 100 to 300 nmol L^−1^ (Fig. 9). The subsequent sampling and analysis of an ice core from the same sopki in March 2018 showed that its methane content had significantly decreased since January (Fig. 9). We also collected samples of ice from shore areas surrounding the sopki near Pereyomnaya River and from ice hills without holes. Throughout their thicknesses, ice cores gave methane concentrations of about 86 nmol L^−1^ (1.93 ppm), which is close to equilibrium with the atmosphere.

## Discussion

This paper describes the first evidence of methane hydrates emerging to the surface of Lake Baikal and validated by photo and video records. The summer detection of hydrates was apparently associated with disturbance of hydrate-rich sediment by a rosette sampler. In winter, we observed methane hydrates ascend spontaneously from the sediment to the lake surface over an entire observation period of several hours. The release was indirectly confirmed by hydroacoustic data showing objects rising with velocities of about 40 cm s^−1^. This speed exceeded typical speeds of concurrently registered gas bubbles by a factor of two. McGinnis *et al*.^8^ summarized experimental observations to show that ascent velocities for bubbles of different sizes do not exceed 35 cm/s. While velocities over 40 cm/s could be theoretically achieved by large bubbles (more than 35 mm diameter), these structures are unstable and quickly dissociate into smaller bubbles. The rapidly ascending objects may represent hydrate coated bubbles which can be formed in Lake Baikal^42^, but simple calculations using Stoke’s Law indicate that they would need to be about 6 mm in diameter. However according to McGinnis *et al*.^8^, a complete hydrate rim forms around bubbles 2.7–4 mm in diameter and bubbles with larger sizes cannot be completely covered by a hydrate rim, and therefore, cannot reach such speeds. Also, detection of these objects at depths shallower than the gas hydrate stability zone suggests that they should already be dissolved. Velocity and depth estimates provided by mathematical modelling matched values observed in echogram data to support this interpretation. Echograms show the objects in question appearing at depths of 250 m (Figs. 3b, 4a) which approximately matches the depth of ice crust formation on hydrates (see Supplementary Fig. S1). Because the reflectivity of an object depends on the ratio of the speed of sound in its material and in the environment, hydrate fragments do not efficiently reflect sound signals. The insonification wavelength of the echosounder was about 5 cm (for 28 kHz), a length comparable to the estimated diameter of ascending methane hydrates. Therefore, their acoustic reflection will not satisfy laws of geometric optics, so the bodies will scatter acoustic waves in different directions. Incident waves may also bend around these objects due to diffraction. Thus, the relatively small size of the ascending bodies and their low reflection coefficients make them difficult to perceive with echosounder detection. By contrast, gas bubbles represent effective reflectors, especially if their diameter corresponds to the resonant radii for the characteristic insonification frequency and depth^8^. Calculations following Clay and Medwin^43^ showed that at 250 m depth and a 28 kHz insonification frequency, the typical resonant size for methane bubbles ranged from 0.3 to 0.6 mm. An ice crust (saturated with small gas bubbles) will began to form around a rising gas hydrate fragment at the depth where the temperature of the hydrate phase transition becomes equal to or less than the freezing point of water. The small bubbles trapped within the ice crust thus becomes a highly efficient reflector. These conditions allow for consistent hydrate detection on echograms. Gas inclusions can also increase hydrate buoyancy and corresponding ascension rate (Fig. 4b), in contrast to bubbles, which usually dissolve and slow down while rising. These principles support the interpretation that ascending objects detected were methane hydrates. Methane hydrates likely disappear in echogram data at 50–70 m due to displacement by the mean current, whose vertically averaged value was 1.2 cm s^−1^. Average lateral displacement of an ascending object by this current would reach 12 meters, a width that exceeds the echosounder’s 10 m field of view at 50 m depth.

We observed ascending methane hydrates in winter using a stationary echosounder installed on the ice surface. Successful detection with this approach demonstrated the unlikelihood of making such observations from a research vessel (RV). Vessel drift (0.3–1 m s^−1^) and especially its usual speed (3–5 m s^−1^) combined with hydrate ascension rates would not allow features to remain in the echosounder field of view for the necessary detection interval. The probability of observing gas hydrates reaching the surface from an RV (Fig. 3a) is also quite small, because the travel time of the released gas hydrates from a depth of 500–1000 m to the surface is about 20–40 min. This time is typically much longer than the retrieval time of instruments. Usually, immediately after retrieving instruments, the RV proceeds directly to the next station. This, along with normal RV drift, makes visually observing hydrates reaching the surface very rare.

Modelling results suggest a hydrate fragment must be at least 2.5 cm in radius when it leaves the upper stability boundary in order to reach the surface of Lake Baikal. This diameter is considerably less (by a factor of four) than previously reported minimum size estimates for hydrates ascending through the marine water column (about 10 cm)^26,27^. This relatively small minimum size required for ascending bodies along with the low probability of methane hydrate detection suggests that in Lake Baikal hydrates can reach the surface quite often but undetected. These results also suggest a direct pathway between sediment derived methane and surface concentrations with subsequent emissions to the atmosphere. This route has not been previously considered in the methane budget of Lake Baikal^7^.

Detection of methane hydrates ascending to the lake surface also supports our hypothesis that gas released during the decomposition of gas hydrates in the water column may contribute to local upwelling of deep waters and generation of circular anticyclonic currents^23,44^. Data obtained from the water column in the area of the ring structure on the lake ice near cape Krestovskiy in June 2017 showed local decreases in the concentration of dissolved ions and water temperature^44^ probably due to decomposition of methane hydrates.

Appearance of methane hydrates at the lake surface during ice cover formation may generate unique structures in the ice. Previous research^45^ has described the anomalous effect of self-preservation of gas hydrates at atmospheric pressure and temperatures below the freezing point of water (240 K ≤ T ≤ 273 K). This preservation is expressed in the long existence (up to several months) of the hydrate remnants in metastable form without significant further decomposition. Observations confirm the formation of protective ice coating around hydrates during their dissociation but the mechanism for ice formation remains poorly understood. We hypothesized that spherical ice morphology (kolobovnik) in pelagic areas of the lake forms due to ice encapsulation of hydrate fragments. Ice formation is mediated by heat absorption during hydrate decomposition (phase transition). When the water temperature is close to zero, discrete ice spheres can freeze together and form a field^33^ or be washed up on the land as fast ice^29^. Analysis of kolobovnik ice cores detected methane concentrations in upper ice layers that greatly exceeded atmospheric concentrations. The atmosphere is thus not a plausible source of methane observed in ice. Elevated methane concentrations beneath winter ice cover^7^ (methane accumulation during limited gas exchange with the atmosphere) do not suffice to explain the high concentrations observed in kolobovnik ice. Additionally, concentrations observed in the ice could not be supplied by gas bubbles because the majority of these dissolve into the Lake Baikal water column^7^ before reaching the surface. Only the much more stable hydrates could deliver the methane volumes observed in ice. The presence of minor soil particles and other detritus within the kolobovnik (Fig. 5b) indicate lake bottom sediments entrained and transported by methane hydrates. Locations of kolobovnik ice are in good agreement with the location of ice openings associated with gas seeps^46^.

Sopki also represents a form of ice likely related to the preservation and decomposition of methane hydrates transported to the lake surface (Fig. 9, Supplementary Fig. S3). We propose that sopki form from hydrate fragments washed ashore by wave action during periods of intense wind. Splash zone water and falling snow freeze on the sopki structure due to low air temperatures (below 0 °C) and heat absorption by hydrate decomposition. The observed hillocks then become partly hollow inside (Fig. 9) due to complete decomposition of initiating gas hydrate. Similar hilly structures without openings contain methane concentrations that approximate atmospheric methane concentrations. The lower methane concentrations in sopki ice relative to those observed in kolobovnik (Fig. 7) may reflect gradual release to the atmosphere through the observed openings, while methane in kolobovnik is preserved by a solid ice cover. Comparison of methane concentrations in January and March 2018 from the same sopki (Fig. 8) feature support this interpretation. Methane concentrations at different sites and from different structures also generally confirms the interpretation that elevated methane concentrations due to methane hydrate decomposition could cause to the formation of observed ice features. At present, there is no other satisfactory explanation for the morphology and elevated methane concentration observed in sopki and kolobovnik ice.

## Conclusions

This study provides novel evidence that methane hydrates released from sediment at 500–1000 m depths can reach the surface of Lake Baikal. Field observations and model results suggest that hydrates exceeding 2.5 cm in radius can ascend and contribute methane to the atmosphere. Current estimates for Lake Baikal methane release assume only diffusion and ebullition exchange processes, and that most sedimentary methane oxidizes within the water column^7^. Methane hydrates ascending to the surface may provide an additional mechanism for methane release into the atmosphere in Lake Baikal and probably in the marine environments, but their contribution requires further investigation. Paull *et al*.^47^ have considered the possibility of methane transport by hydrates released by marine slumping and rising through the ocean water column. Kvenvolden^48^ in turn, interprets these scenarios in terms of geological/climatic risk.

This report provides novel *in situ* detection of the methane hydrates randomly ascending from bottom sediments of a deep lake. Hydroacoustic properties of the hydrate fragments make them difficult to detect in the water column beneath the depth of ice crust formation. At shallower depths, however, they may be obscured behind a large number of accompanying bubbles, since they have comparable target strength. This could be the main reason that no one has observed them before. Specialized long-term and large-scale studies on the frequency of such events in Lake Baikal and in marine environments can further help to quantify the contribution of this methane hydrate transport mechanism.

Unique ice morphologies and structures found around Lake Baikal likely form due to methane hydrates ascending from lake sediment. Analysis of gas inclusions from the ice structures revealed elevated methane concentrations relative to atmospheric values and relative to those measured from surrounding ice. While sopki typically appear on an annual basis, kolobovnik occurs only rarely in pelagic areas of the lake. A study of interannual variation in the distribution of these ice forms can help elucidate long term spatial and temporal patterns in methane hydrate transport and release. The unique kolobovnik and sopki morphologies arise from environmental conditions and physical mechanisms that contribute to their formation. Detailed observations can further clarify the processes occurring during the earliest stages of kolobovnik and sopki formation.

## Supplementary information

Supplementary Information

## Data Availability

All data generated or analysed during this study are included in this article and its Supplementary Information file. Graph data or other information can be requested via email to nick@lin.irk.ru.
