# Supplementary material for: Methane hydrate emergence from Lake Baikal: direct observations, modelling, and hydrate footprints in seasonal ice cover
Source: Sci Rep. 2019 Dec 18;9:19361. doi: 10.1038/s41598-019-55758-8 (PMC6920351; doi:10.1038/s41598-019-55758-8)
Supplement: Supplementary file 1 — Supplementary Information [file 41598_2019_55758_MOESM1_ESM.pdf]

Methane hydrate emergence from Lake Baikal: direct observations, modelling, and hydrate footprints in seasonal ice cover

N. G. Granin[1], I. A. Aslamov[1], V. V. Kozlov[2], M. M. Makarov[1], G. Kirillin[3], D. F. McGinnis[4,*], K. M. Kucher[1], V.V. Blinov[1], V. G. Ivanov[1], I. B. Mizandrontsev[1], A. A. Zhdanov[1], A. S. Anikin[2], M. N. Granin[2], R. Yu. Gnatovsky[1]

[1]Limnological Institute, Siberian Branch of Russian Academy of Science, (LIN SB RAS) Irkutsk, 664033, Russia
[2]Matrosov Institute for System Dynamics and Control Theory of Siberian Branch of Russian Academy of Sciences (ISDCT SB RAS), Irkutsk, 664033, Russia
[3]Leibniz-Institute of Freshwater Ecology and Fisheries (IGB), Berlin, 12587, Germany
[4]Aquatic Physics Group, Department F.-A. Forel for Environmental and Aquatic Sciences (DEFSE), Faculty of Science, University of Geneva, Geneva, 1211, Switzerland

[*]daniel.mcginnis@unige.ch

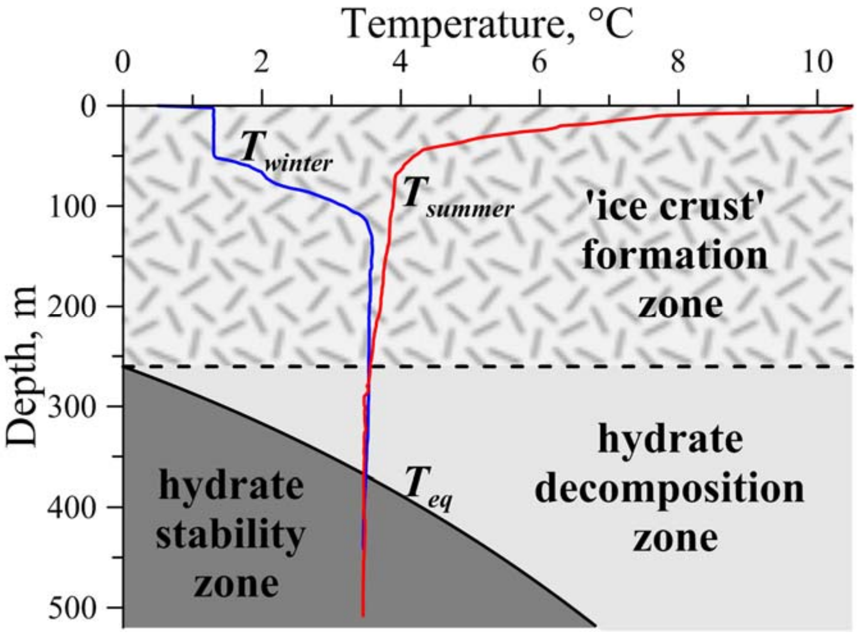

Figure S1. Plot of in situ temperatures in Lake Baikal at the Gas seep "Stupa" obtained on 28 July 2013 (T_{summer}) when the emergence of gas hydrates at the lake's surface were observed and on 5 April 2013 (T_{winter}) when echograms with fast uprising objects were obtained; T_{eq} is the temperature–depth boundary of stability of gas hydrates Structure I. Plot illustrates where gas hydrate is stable, where its decomposition begins, and where "ice crust" formation begins.

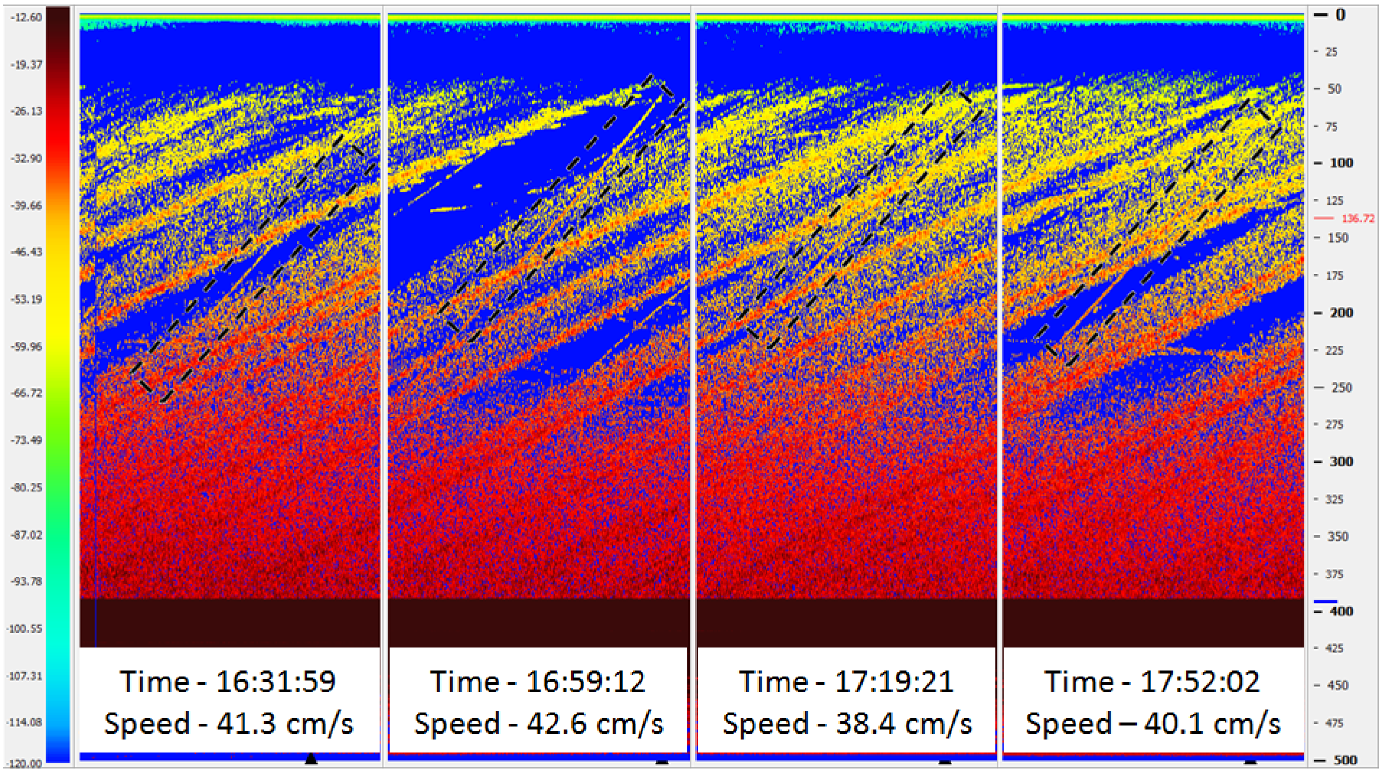

Figure S2. A series of echograms with fast uprising objects. Gas seep "Stupa", 5 April 2013.

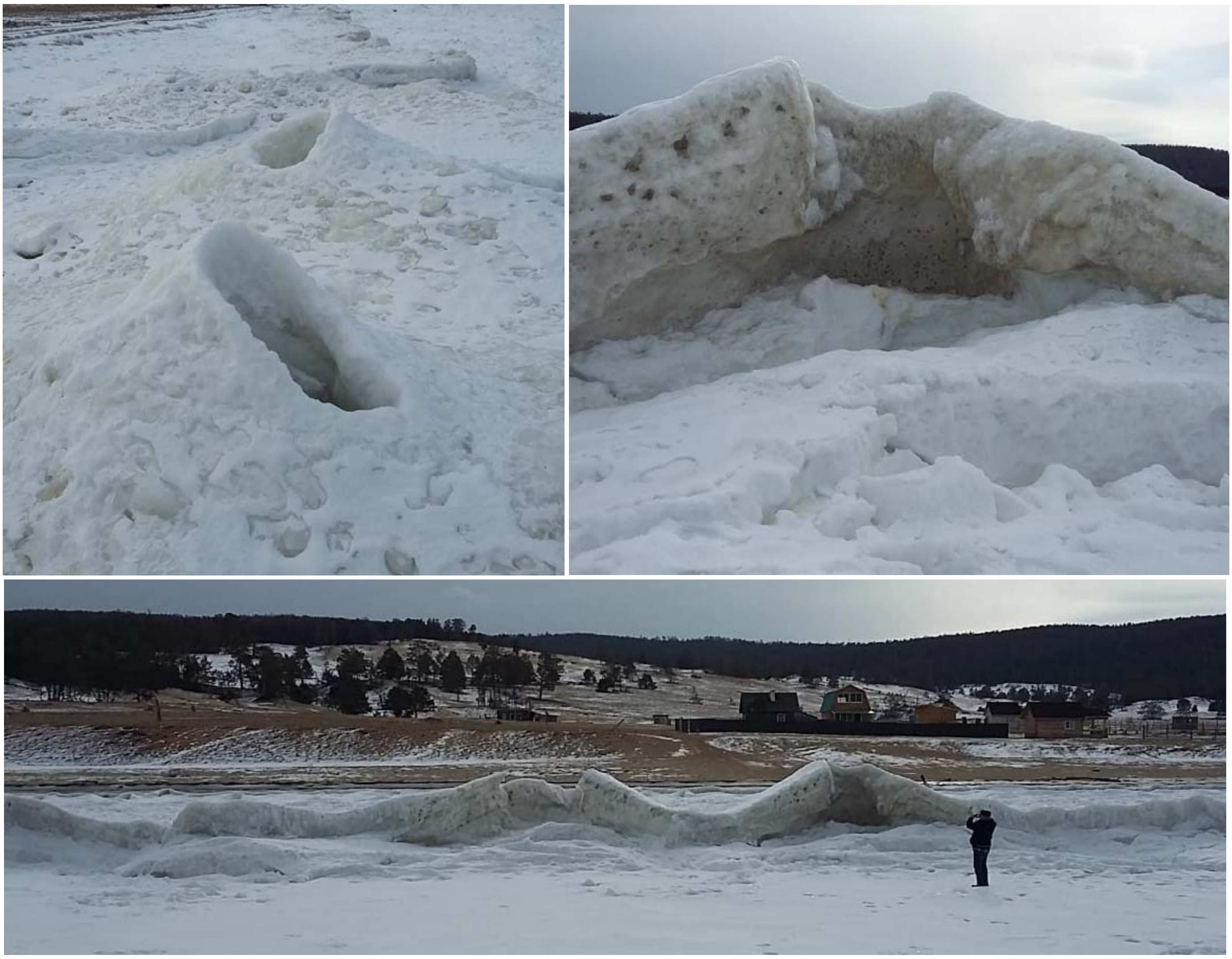

Figure S3. A photograph of a sopki on the western shore of Olkhon island taken in the end of February, 2019.

Methods. Model of the gas hydrate rising

Phase transition

Decomposition of a gas hydrate with an equivalent spherical radius a_h is driven by convective heat transfer across the "ice crust" of thickness $\delta_i = a_i - a_h$, where a_i is the equivalent spherical radius of the outside border of the "ice crust". The inner border of the "ice crust" is the same as the outer border of the gas hydrate a_h. For the gas fraction with density ρ_g we utilize the equation of state in form $p = \rho_g^0 R_g T$ and assume that the structure of the "ice crust" has negligibly small diffusion resistance during the passage of gas through it. Then, the change in mass of a gas hydrate with an "ice crust" in the process of decomposition at temperatures below the freshwater freezing point, T<273.15 K, will only occur as a result of the supply of heat from the environment, i.e. the decomposition process is limited by the supply of heat to the surface of the gas hydrate only. Further, we assume the temperature on the hydrate-"ice crust" boundary is equal to an equilibrium value of phase transformation for the gas hydrate at current values of hydrostatic pressure, whereas temperature on the "ice crust"-water boundary is equal to the freezing temperature of water. The effects of decomposition associated with the diffusion of heat inside the hydrate, and the "self-preservation" (kinetics of slow decomposition) of gas hydrates are neglected[1,2].

The hydrostatic pressure changes with the motion of the hydrate along the vertical axis z as

$$p = p_a + \rho_l g(H_0 - z)\,, \quad \text{(S1)}$$

where z is directed upward with its origin at the initial depth H_0 somewhere below the upper limit of stability of gas hydrates in Lake Baikal, p_a is the atmospheric pressure at the level of the Lake Baikal, g is the acceleration due to gravity and ρ_l is the density of water. For the decomposition of gas hydrates at temperatures > 273.15 K into the gas and water, the equilibrium temperature of the phase transformation T_f is determined as

$$T_f(p) = T_{h0} + T_* \ln(p / p_{h0}) \quad \text{(S2)}$$

where: $T_{h0} = 278.0\,\mathrm{K}$ is the equilibrium temperature, corresponding to the pressure value $p_{h0} = 4.28 \cdot 10^6\,\mathrm{Pa}$, $T_* = 9.91\,\mathrm{K}$ - is an empirical parameter[1]. For the decomposition of gas hydrates at temperatures <273.15 K, the gas-ice formula

$$\ln(p) = 10.075 - 2750.3 T_f^{-1} + 69345 T_f^{-2} \quad \text{(S3)}$$

is used, where p is measured in Mpa[3].

Rising velocity

The equation for determining the ascending speed of gas hydrates $w(t)$ with a spherical shape and an "ice crust" is obtained from the equation of impulses for a body with variable mass:

$$(m_h + m_w)\frac{dw}{dt} = f_A - f_T - f_W - f_M\,, \quad \text{(S4)}$$

where, m_h is mass of gas hydrate, m_w – joined mass of water,

$$f_A = \frac{4}{3}\pi a_i^3 \rho_l g, \quad f_T = \frac{4}{3}\pi a_i^3 \bar{\rho}_h g, \quad f_W = c_w \pi a_i^2 \frac{\rho_l w^2}{2}, \quad f_M = w\frac{d(m_h + m_w)}{dt}, \quad \text{(S5)}$$

f_A, f_T, f_W, f_M are buoyancy, gravity, hydrodynamic resistance and reactive force, respectively. $m_h = \frac{4}{3}\pi a_i^3 \bar{\rho}_h$ – is the mass of gas hydrate with "ice crust". $m_w = \frac{2}{3}\pi a_i^3 \rho_l$ – is the attached mass for a sphere[4]. We define the density of porous ice $\rho_i = (1-n)\rho_i^0 + n\rho_g^0$, where ρ_i^0 – is the density of monolithic ice. The porosity of the ice formed during the decomposition of gas hydrate is assumed to be equal to $n = 0.134$ (number of hydration 6.0). The average density of the hydrate ρ_h with "ice crust" is equal to $\bar{\rho}_h = \rho_h \frac{a_h^3}{a_i^3} + \rho_i \frac{(a_i^3 - a_h^3)}{a_i^3}$. The hydrodynamic drag coefficient c_w is calculated using the formula[5,6,7] which accuracy is not worse than 6% in a wide range of Reynolds numbers ($Re < 10^5$).

$$c_w = \frac{24}{\mathrm{Re}}(1 + 0{,}15\,\mathrm{Re}^{0{,}687}) + 0{,}42(1 + 42500\,\mathrm{Re}^{-1{,}16})^{-1}, \quad \mathrm{Re} = \frac{2a_i \rho_l w}{\mu_l}, \quad \text{(S6)}$$

where: μ_l – dynamic viscosity of water, depending on water temperature T_l.

The current coordinate of position of the hydrate was determined from the velocity of the gas hydrate vertical motion as $\frac{dz}{dt} = w(t)$ with the initial values $z_0 = 0.0\,\mathrm{m}$ and $w_0 = 0.001\,\mathrm{m\,s^{-1}}$.

Decomposition rates

The equations for determining the radius of gas hydrate $a_h(t)$ and the radius of the hydrate "ice crust" $a_i(t)$ were determined from the heat balance at the boundaries of phase transformations of hydrate-"ice crust" and "ice crust"-water. We considered three characteristics stages of the rise from depth H_o = 500 m.

1. The ascent from the initial depth to the upper boundary of stability ($T_{fh} \geq T_l$). Here, the speed of the stationary uniform rise was determined without considering the processes of dissolution, due to their insignificance in the zone of stability of gas hydrates[5].

$$\frac{da_i}{dt} = 0, \; \frac{da_h}{dt} = 0. \tag{S7}$$

2. Rising in the hydrate-gas-water phase ($T_{fi} \leq T_{fh} < T_l$). At this stage, thickness of the "ice crust" was $\delta_i = 0$, so that,

$$\frac{da_i}{dt} = 0, \quad \rho_h l_{hl} \frac{da_h}{dt} = q_l - q_h, \quad q_l = k(T_{fh} - T_l), \quad q_h = 0. \tag{S8}$$

3. Ascent in the range of temperatures where the hydrate-gas-ice exists ($T_{fh} < T_{fi}$). The thickness of the "ice crust" $\delta_i > 0$, and,

$$\rho_h l_{hi} \frac{da_h}{dt} = q_{hi} - q_h, \quad q_{hi} = \frac{Q_i}{4\pi a_h^2}, \quad q_h = 0. \tag{S9}$$

$$\rho_i l_{il} \frac{da_i}{dt} = q_l - q_{il}, \quad q_{il} = \frac{Q_i}{4\pi a_i^2}, \quad q_l = k(T_{fi} - T_l). \tag{S10}$$

Here, q_l, q_{il} are the densities of heat flow on the water-"ice crust" boundary, and q_h, q_{ih} are those at the "ice crust"-gas hydrate boundary, assumed that $q_{ih} >> q_h$. $k = \frac{Nu_l \lambda_l}{2a_i}$ is the coefficient of heat transfer; λ_l is the thermal conductivity of water; l_{hi}, l_{il}, l_{hl} – the heat of phase transformation of hydrate-ice, ice-water and hydrate-water, respectively[1,3,8,9].

$$Q_i = 4\pi \bar{\lambda}_{ef} \frac{a_h a_i}{a_i - a_h}(T_{fh} - T_{fi}) \tag{S11}$$

is heat flow in a spherical "ice crust" with a linear distribution of the thermal conductivity[10]; T_{fh}, T_{fi} are the temperatures on the boundaries of phase transformations of hydrate-ice and ice-water, respectively; $\bar{\lambda}_{ef} = k_{ef}\bar{\lambda}$ is the average or effective thermal conductivity of the "ice crust". Since the thermal properties of the "ice crust" are not exactly known, we introduced an unknown coefficient k_{ef}, which is later determined from the solution of the inverse problem. The model of thermal conductivity $\bar{\lambda}$ of porous ice saturated with gas, is obtained from the formula of heat transfer for a multilayer spherical wall, which is obtained by integrating values of thermal conductivities of ice $\bar{\lambda}_i$ and gas $\bar{\lambda}_g$ assuming linear change of temperature through the corresponding layer[10]. For the double-layer spherical shell it takes the form

$$\frac{1}{\bar{\lambda}}\left(\frac{1}{a_h} - \frac{1}{a_i}\right) = \frac{1}{\bar{\lambda}_g}\left(\frac{1}{a_h} - \frac{1}{a_g}\right) + \frac{1}{\bar{\lambda}_i}\left(\frac{1}{a_g} - \frac{1}{a_i}\right), \tag{S12}$$

where the radius of the layer of gas is calculated from the ratio: $a_g^3 = na_i^3 + (1-n)a_h^3$.

The Nusselt number Nu_l was calculated according to the formula,

$$Nu_l = 1 + (1 + \Pr \mathrm{Re})^{1/3}\left(1 + \frac{0,096\,\mathrm{Re}^{1/3}}{1 + 7\,\mathrm{Re}^{-2}}\right), \quad \mathrm{Re} < 10^5, \quad \Pr = \frac{\nu_l}{\chi_l} \tag{S13}$$

which is valid in a wide range of Reynolds ($Re < 10^5$) and Prandtl Pr numbers[5]. Here, ν_l, χ_l are the kinematic viscosity and thermal diffusivity depends on the temperature of the water T_l.

Input parameters and solution procedure

The values of density ρ_l, dynamic viscosity μ_l, thermal conductivity λ_l, kinematic viscosity ν_l and thermal diffusivity χ_l for pure water were determined in the temperature range from 0 to 20 °C by linear interpolation of tabular data[11]. During the numerical calculations for thermal properties of gas hydrates, the following values were adopted: $l_{hi} = 18\cdot10^3\,\mathrm{J\,mol^{-1}}$, $l_{il} = 6\cdot10^3\,\mathrm{J\,mol^{-1}}$, $l_{hl} = 54\cdot10^3\,\mathrm{J\,mol^{-1}}$ [1,3]. The thermal conductivity of pure ice was obtained from Bogorodskiy and Gavrilo[8]. The thermal conductivity of methane gas was from Vargaftik[11]. The density of the gas hydrate was taken as $\rho_h = 912\,\mathrm{kg\,m^{-3}}$. The atmospheric pressure at an altitude of 456 m (the level of Lake Baikal) was assumed to be equal to $p_a = 0.96\cdot10^5\ \mathrm{Pa}$.

The obtained closed system of four ordinary differential equations was solved numerically with regard to the variables $z(t), w(t), a_h(t), a_i(t)$. The direct problem was solved by the Runge-Kutta method with the initial values $z(t)_{t=0} = z_0$, $a_h(t)_{t=0} = a_h^0$, $a_i(t)_{t=0} = a_i^0$, and $w(t)_{t=0} = w_0$, $H_0 = 500.0\ \mathrm{m}$. The inverse problem of identifying unknown parameters – initial radius a_h^0 and the coefficient k_{ef}, were solved from experimental data on rising speeds. Experimental values on the trajectory and speed were taken from the echogram emersion of real objects under the ice cover of Lake Baikal in April 2013 (in the area of the gas seep "Stupa") together with a concurrent a temperature profile. An approximate solution of the inverse problem, was achieved by minimizing a quadratic smoothing of the function of Tikhonov and Arsenin[12] for the vector of unknown parameters.

References

1. Istomin, V. A., and Yakushev, V. S. *Gas Hydrates In Natural Conditions*. (Nedra, Moscow, 1992).
2. Uchida, T., Sakurai, T., and Hondoh, T. Ice-shielding models for self-preservation of gas hydrates. *J. Chem. Chem. Eng.* **5,** 691-705 (2011).
3. Anderson, G. K. Enthalpy of dissociation and hydration number of methane hydrate from the Vernier equation. *J. Chem. Thermodynamics* **36,** 1119-1127 (2004).
4. Batchelor, G. K. *An introduction to fluid dynamics* (Cambridge University Press, Cambridge, United Kingdom, 2000).
5. Zhang, Y., and Xu'll, Z. Kinetics of crystal dissolution and melting, with application to methane hydrate dissolution and dissociation in seawater. *Letters Earth Planet. Sci.* **213,** 133-148 (2003).
6. Clift, R., Grace, J. R., and Weber, M. E. *Bubbles, Drops, and Particles*. (Academic Press, New York, USA, 1978).
7. Kutepov, A. M., *et al. Chemical Hydrodynamics*. (Kvantum, Moscow, Russia, 1996).
8. Bogorodskiy, V. V., and Gavrilo, V. P. *Ice. Physical properties. Modern Methods of Glaciology*. (Gidrometeoizdat, Leningrad, USSR, 1980).
9. Shagapov, V., Tazetdinov, B. I., and Nurislamov, O. R. A contribution to the theory of gas hydrate particle formation and decomposition in the process of their ascent in water. *Vestn. Tomsk. Gos. Univ. Mat. Mekh.* **6(26),** 106-113 (2013).

10. Tsvetkov, F. F., and Grigoriev, B. A. *Heat and Mass Transfer: a Textbook for Higher Educational Institutions*. (Izdatelstvo MEI, Moscow, Russia, 2005).
11. Vargaftik, I. B. *Handbook on Thermophysical Properties of Gases and Liquids*. (Nauka, Moscow, USSR, 1972).
12. Tikhonov, A.N., and Arsenin, V.Y. *Solutions of Ill-Posed Problems*. (Winston and Sons, Washington DC, USA, 1977).
